# Catastrophic intraoperative failure of a ceramic femoral head

**DOI:** 10.1016/j.artd.2021.08.017

**Published:** 2021-10-08

**Authors:** Paul Dobria, Arpan Patel, Brett Levine

**Affiliations:** a Chicago Medical School, Rosalind Franklin University of Medicine and Science, North Chicago, IL, USA; b Department of Orthopedics, Rush University Medical Center, Chicago, IL, USA

**Keywords:** Ceramic femoral head fracture, Revision total hip arthroplasty, Titanium adapter sleeves, Metallosis, Trunnionosis

## Abstract

Approximately 17 years after a primary metal-on-metal total hip arthroplasty, a 59-year-old female developed pain, swelling, and weakness in her right hip accompanied by laboratory findings and imaging suggestive of an adverse local tissue reaction. Acetabular revision was performed to upsize the femoral head and improve hip stability. Upon impaction of the new, non-option ceramic femoral head onto the unsleeved retained stem, the head split into two pieces without fragmentation. The surgery was completed using a cobalt-chromium head, which was impacted without issue onto the stem’s taper. Although BIOLOX delta femoral heads do not require titanium sleeves, we believe that careful consideration should be given to their use in revision total hip arthroplasty with ceramic heads, regardless of the extent of trunnion damage noted intraoperatively.

## Introduction

Since their inception, ceramic bearing components have been increasingly used in total hip arthroplasty (THA) [1,2]. Ceramic bearings are heralded for their biological inertness, wettability, scratch resistance, improved wear characteristics, and decreased infection rates relative to metal-on-metal (MoM) and metal-on-polyethylene (MoP) components [[2], [3], [4], [5]]. These wear characteristics, biomaterial properties, and resistance to trunnion damage have made their use desirable, especially for younger patients who wish to maintain an active lifestyle [1,2,6]. Furthermore, recent concerns for trunnionosis with cobalt-chromium femoral heads have driven a shift toward using ceramic heads in THA, with utilization reported in over two-third of primary THAs performed in the United States [2].

Unlike their metallic counterparts, inherent to ceramic femoral heads is a higher modulus of elasticity and an associated increased risk of fracture, [[7], [8], [9]] although compositional improvements among successive generations of ceramic components have progressively decreased this risk [[10], [11], [12], [13], [14], [15]]. Most recently, compositional optimization of fourth-generation BIOLOX delta ceramic heads (BIOLOX delta; CeramTec, Plochingen, Germany) to include nano-sized, yttria-stabilized tetragonal zirconium and strontium oxide has resulted in a reduction of fracture rates to 0.003%, down from the 0.021% rate seen with alumina ceramic heads [10,16]. Despite these advances, fracture of ceramic components nonetheless represents a catastrophic complication necessitating surgical revision with limited reconstructive options [10].

**Figure 1:**
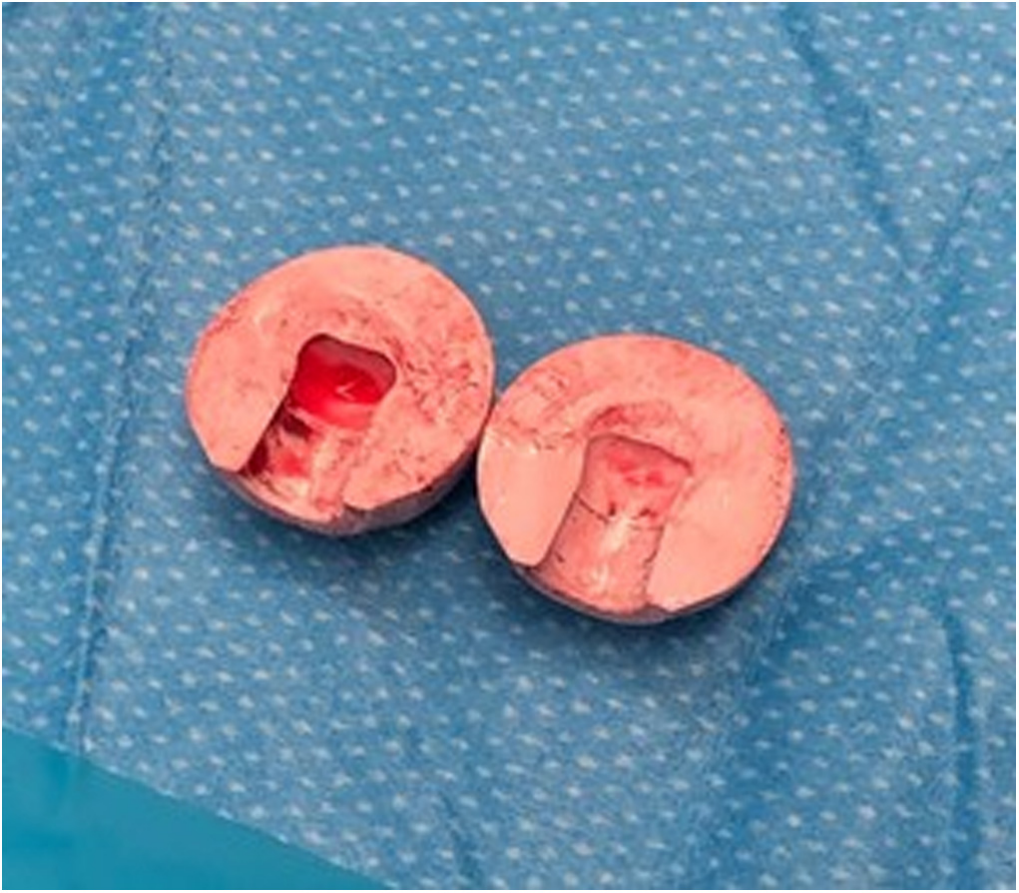
Replacement BIOLOX delta ceramic femoral head, split into two large pieces without fragmentation after impaction onto the native femoral head.

This case report describes an intraoperative fracture of a BIOLOX delta ceramic femoral head (CeramTec, Plochingen, Germany) during impaction onto a well-fixed femoral stem as part of a revision THA after MoM failure of the initial implant at 17 years postoperatively (Fig. 1). To our knowledge, this is the first instance of such a fracture occurring intraoperatively; in all other published case reports, fracture occurred as a postoperative complication (Table 1). The patient described in this case was informed that pertinent information regarding her case would be used for publication and provided consent.

**Table 1:** Reported cases of fractured ceramic femoral heads.

Reporting author	Year	Femoral head diameter (mm)	Liner material	No. of cases	Mechanism of injury
Howard et al. [12]	2017	32	Ceramic	3	Not reported
Howard et al. [12]	2017	34	Ceramic	4	Not reported
Heiner and Mahoney [17]	2017	36	Ceramic	1	Bicycle accident
Lombardi et al. [18]	2009	28	Ceramic	1	Rose from commode
Pomeroy et al. [9]	2015	32	Polyethylene	1	Atraumatic
Tucker and Acharya [19]	2013	28	Polyethylene	1	Rose from bed
U.S. FDA [20]	2011	36	Not reported	1	Fall on ice
U.S. FDA [20]	2015	32	Not reported	1	Fall
U.S. FDA [20]	2015	36	Not reported	1	Fall
U.S. FDA [20]	2016	36	Not reported	1	Injury
U.S. FDA [20]	2016	28	Ceramic	1	Injury
U.S. FDA [20]	2016	28	Not reported	1	Injury
U.S. FDA [20]	2017	Not reported	Not reported	1	Fall
U.S. FDA [20]	2017	Not reported	Not reported	1	Fall
U.S. FDA [20]	2017	36	Not reported	1	Bicycle accident
U.S. FDA [20]	2017	36	Not reported	1	Injury
U.S. FDA [20]	2018	36	Not reported	1	Injury

## Case history

A 59-year-old female underwent staged bilateral primary THA, with procedures performed within 1 year of each other. In each case, a posterolateral approach was used, and she received a dual modular implant with an MoM articulation. The femoral component was an 11/13 taper titanium dual modular implant, and the acetabulum was a titanium modular cup (S-ROM and Pinnacle; DePuy, Warsaw, IN). Both procedures were uncomplicated. At approximately 17 years postoperatively, the patient reported severe pain in her right hip accompanied by swelling, numbness, and weakness. She additionally complained of new-onset constitutional symptoms including malaise and lightheadedness. Physical examination revealed a right-sided Trendelenburg gait.

**Figure 2:**
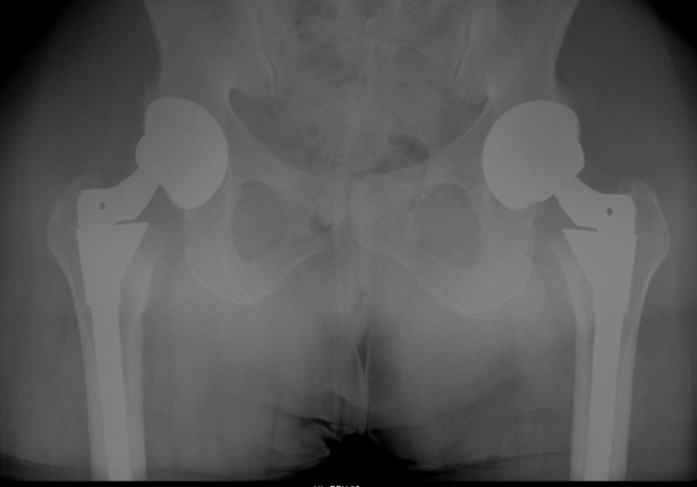
Preoperative radiograph obtained at the time of patient’s initial presentation. All arthroplasty components are in acceptable position without evidence of wear or loosening.

**Figure 3:**
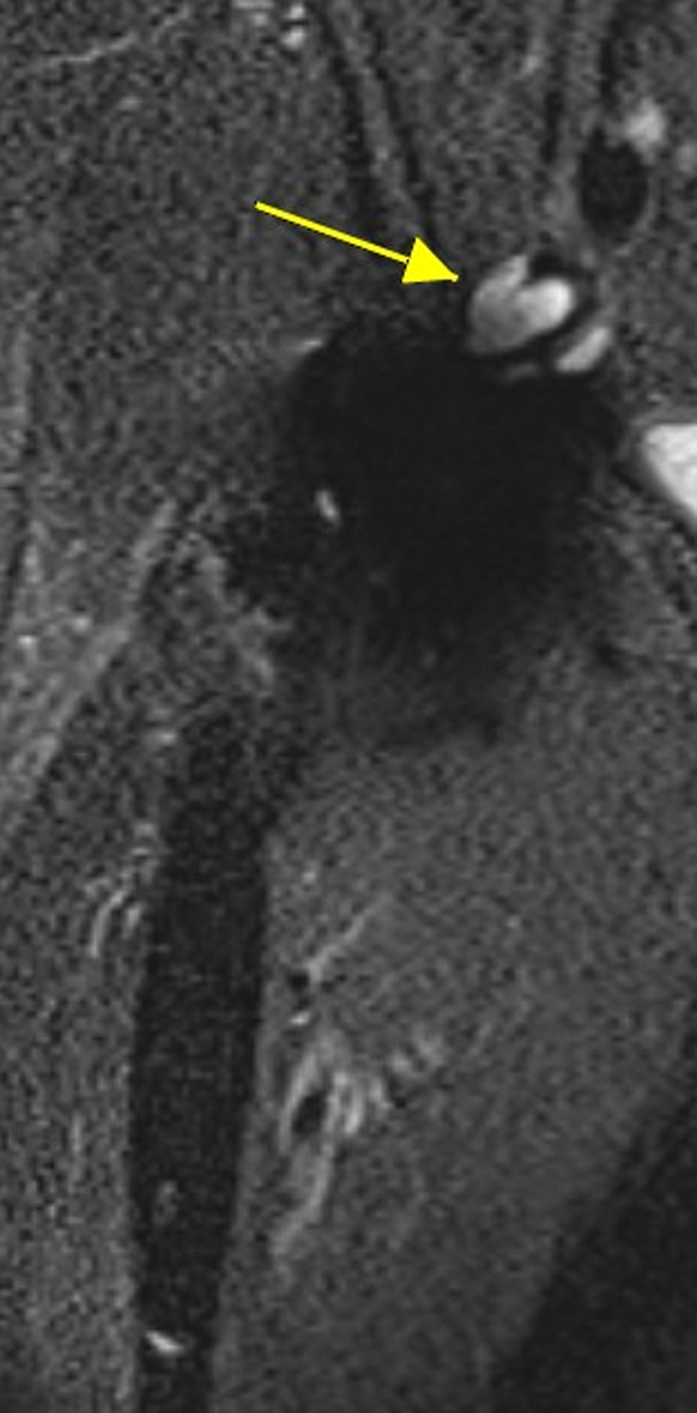
metal artifact reduction sequence MRI revealing fluid collection (yellow arrow) in the anterior aspect of the right hip extending into the iliopsoas bursa. Coronal short tau inversion recovery setting.

Radiographs obtained at the time of presentation revealed all arthroplasty components in their acceptable position without evidence of osteolysis, metal debris, or component wear or loosening (Fig. 2). Laboratory testing revealed markedly elevated serum levels of both cobalt (49.7 μg/L) and chromium (22.5 μg/L). In addition, a metal artifact reduction sequence MRI revealed fluid collection in the anterior aspect of the right hip extending into the iliopsoas bursa (Fig. 3). At this point, the decision was made to proceed with revision surgery, planning for a minimum of head/liner exchange with further assessment of the native cup and stem.

Intraoperatively, surgical visualization revealed a mechanically stable implant without evidence of trunnion damage. The cup and the femoral sleeve were ingrown, and the modular stem was well fixed to the femur. Internally, there was a mild adverse local tissue reaction with preservation of the abductors.

Upon atraumatic removal of the 36 mm, +0 cobalt chromium femoral head, the trunnion was noted to be visually intact and undamaged with minimal corrosion [21]. Initial efforts to remove the native femoral stem from within the modular sleeve were unsuccessful. With no visible debris coming from either taper, the existing pathology was attributed to the MoM articulation, and the stem was retained.

The synovium was debrided of necrotic debris and thickened tissue. In the interest of revising the native vertical positioning and aiding with head size options and hip stability, the 50-mm acetabular cup was removed atraumatically with a curved osteotome system and revised to a size 56-mm modular titanium acetabular component (Pinnacle; DePuy, Warsaw, IN) and a 40-mm, face-changing, +4-mm offset polyethylene liner. After trialing, it was noted that a unique (11/13) taper +12-mm, 40-mm head offered good stability, equal limb lengths, and restoration of her offset.

The trunnion was then irrigated with pulsatile lavage, dried, and prepared to accept the new femoral head—a 40-mm, +12-mm BIOLOX delta ceramic head (CeramTec, Plochingen, Germany). A ceramic head was then placed without a sleeve on what appeared to be an undamaged trunnion. The in situ femoral stem has a unique taper for which titanium sleeves or “option” ceramic heads are not currently available. Given the clean, undamaged trunnion, it was decided to proceed with a standard ceramic femoral head. Upon single-blow impaction of the new head onto the stem, the head split into two large pieces without fragmentation (Fig. 1). Fifty percent of the head fell into the cup, and the remainder stayed attached to the femoral neck, requiring extraction. Although the extraction itself merely involved tapping the remainder of the femoral head off the femoral neck and was by no means arduous, the antecedent fracture implied the presence of either an intrinsic defect of the femoral head or a surface defect of the trunnion. Rather than further attempting a stem removal or risking the possibility of a repeat, potentially comminuted intraoperative fracture, a cobalt chromium head was used to complete the revision. A 40-mm, +12-mm cobalt-chromium head was used and impacted without issue onto the stem’s Morse taper.

The patient had an uneventful postoperative course. At 6-month follow-up, she was ambulating unassisted and pain free, and postoperative radiographs were satisfactory. Laboratory analysis revealed a decrease in both serum cobalt (1.8 μg/L) and chromium (6.5 μg/L) levels to normal reference ranges, accounting for the presence of a preexisting left THA with an MoM articulation. These will be rechecked at 1-year follow-up and, if normal at that time, biennially.

## Discussion

Although rare, ceramic head fractures represent a catastrophic surgical complication [10]. Ceramic head fractures are thought to arise from a combination of factors, including postoperative trauma, suboptimal surgical technique, component defects or incompatibility, deformation of the femoral stem taper, and exceeding the accepted manufacturer implant tolerances [[8], [17], [18], [22]]. To date, all reported instances of ceramic head fractures have arisen as postoperative complications, predominantly as traumatic events (Table 1).

Of interest in this case is a ceramic head fracture arising from potentially unrecognized trunnion damage at the femoral head/neck taper connection upon insertion of a new femoral head. Trunnion damage has been implicated as a mechanism for ceramic component failure, [23], [24] thought to arise from fretting and corrosion at the interface between the femoral stem and head [[25], [26], [27]]. However, this mechanism of fracture classically manifests as a postoperative complication rather than intraoperatively with a clean split (Table 1). Given the normal intraoperative appearance of the trunnion as well as the patient’s elevated preoperative serum metal levels, we believe that the fracture most likely occurred as a consequence of the vertically positioned acetabular cup—leading to edge loading at the MoM articulation between the native femoral head and cup—rather than due to occult trunnionosis.

Nonetheless, trunnionosis, if present at the time of revision, does not necessarily require removal of the original femoral stem [28]. When possible, a well-placed femoral stem ought to be retained to prevent intraoperative complications and reduce recovery time [29]. Whether or not the femoral stem can be retained, however, is dictated by the condition of the trunnion at the time of surgery [30]. A trunnion with excessive corrosion may not accept a new ceramic femoral head [30], and placement of a ceramic head on a suboptimal stem taper surface may, through the creation of nonuniform contact, result in significantly increased tensile stresses [31], [32]. Owing to their inherently brittle nature, ceramic components are susceptible to rapid crack dissemination, particularly when a trauma exceeds their tensile strength [33]. While this property of ceramic components is well known, it is unlikely that it would manifest intraoperatively.

In this case, although the trunnion did initially appear undamaged, a minor defect could have gone unnoticed, as such tolerance variance may be too fine to see with the human eye [32]. In addition, unintended contact between the stem taper and the tool used during ball-head exchange may have created a surface defect or introduced a contaminant onto the stem taper, creating a focal area of increased stress on the ceramic head, predisposing it to fracture [32]. The trunnion in question is smooth but of smaller diameter than the standard 12/14 taper of most modern hip arthroplasty stems. A smooth trunnion has been shown to be favorable for providing better fixation and reducing volumetric wear rates [34]. Surface topography, time in situ, trunnion length, and material can all impact trunnion damage seen at the time of revision. Owing to the inability to pinpoint an exact cause of trunnion damage, it may be best to assume the presence of damage in all cases and use a titanium-sleeved ceramic head [31]. The fractured femoral head was sent to the company for further evaluation; despite multiple attempts to obtain pictures and a further analysis of the head, as of the writing of this article, the company has not responded to our queries.

We believe that this intraoperative fracture could potentially have been prevented with the use of a titanium alloy sleeve, which would have helped recreate a smooth, uniform surface with which the femoral stem taper could articulate [35]. A significant body of research suggests the utilization of a titanium-sleeved ceramic component to be ideal for revision THA, particularly in the settings of trunnion damage, mechanically assisted crevice corrosion, and for cases with associated adverse local tissue reaction [35], [36]. Their use has been reported to be safe, with retrieval studies showing minimal subsequent fretting or corrosion [29], [37]. While the use of a titanium-sleeved ceramic head is not guaranteed to prevent implant fracture, damage not visible to the human eye can cause improper seating and/or loss of contact area between the internal aspect of the femoral head and the trunnion, increasing the risk for ceramic fracture either at the time of impaction or postoperatively [31]. The BIOLOX delta system (CeramTec, Plochingen, Germany) offers a titanium sleeve option that is compatible with their ceramic heads. However, this adapter is not yet approved for use in the United States with our patient’s native femoral stem taper.

## Summary

In addition to sparing the need for invasive and potentially complicated stem revisions, titanium adapter sleeves have as their primary benefit the reduction of tensile stresses in newly implanted ceramic femoral heads [35]. Therefore, although BIOLOX delta heads do not require their use, we believe that consideration should be given to the use of titanium sleeves in revision THA with ceramic heads. In fact, impaction of a ceramic head on a used trunnion without a titanium adapter sleeve should be considered a relative contraindication, regardless of the visual damage score that is noted intraoperatively.

## Informed patient consent

The patient described in this case was informed that pertinent information regarding her case would be used for publication and provided consent.

## Supplementary data

Conflict of Interest Statement for Dobria

Conflict of Interest Statement for Levine

Conflict of Interest Statement for Patel
